# Zika Virus Subverts Stress Granules To Promote and Restrict Viral Gene Expression

**DOI:** 10.1128/JVI.00520-19

**Published:** 2019-05-29

**Authors:** Gaston Bonenfant, Nina Williams, Rachel Netzband, Megan C. Schwarz, Matthew J. Evans, Cara T. Pager

**Affiliations:** a Department of Biological Sciences, The RNA Institute, University at Albany-SUNY, Albany, New York, USA; b Department of Microbiology, Icahn School of Medicine at Mount Sinai, New York, New York, USA; Instituto de Biotecnologia/UNAM

**Keywords:** G3BP1, HuR, stress granule, Zika virus

## Abstract

Flaviviruses limit the cell stress response by preventing the formation of stress granules (SGs) and modulate viral gene expression by subverting different proteins involved in the stress granule pathway. In this study, we investigated the formation of stress granules during Zika virus (ZIKV) infection and the role stress granule proteins play during the viral life cycle. Using immunofluorescence and confocal microscopy, we determined that ZIKV disrupted the formation of arsenite-induced stress granules and changed the subcellular distribution, but not the abundance or integrity, of stress granule proteins. We also investigated the role of different stress granule proteins in ZIKV infection by using target-specific short interfering RNAs to deplete Ataxin2, G3BP1, HuR, TIA-1, TIAR, and YB1. Knockdown of TIA-1 and TIAR affected ZIKV protein and RNA levels but not viral titers. Conversely, depletion of Ataxin2 and YB1 decreased virion production despite having only a small effect on ZIKV protein expression. Notably, however, depletion of G3BP1 and HuR decreased and increased ZIKV gene expression and virion production, respectively. Using an MR766 *Gaussia* Luciferase reporter genome together with knockdown and overexpression assays, G3BP1 and HuR were found to modulate ZIKV replication. These data indicate that ZIKV disrupts the formation of stress granules by sequestering stress granule proteins required for replication, where G3BP1 functions to promote ZIKV infection while HuR exhibits an antiviral effect. The results of ZIKV relocalizing and subverting select stress granule proteins might have broader consequences on cellular RNA homeostasis and contribute to cellular gene dysregulation and ZIKV pathogenesis.

**IMPORTANCE** Many viruses inhibit SGs. In this study, we observed that ZIKV restricts SG assembly, likely by relocalizing and subverting specific SG proteins to modulate ZIKV replication. This ZIKV-SG protein interaction is interesting, as many SG proteins are also known to function in neuronal granules, which are critical in neural development and function. Moreover, dysregulation of different SG proteins in neurons has been shown to play a role in the progression of neurodegenerative diseases. The likely consequences of ZIKV modulating SG assembly and subverting specific SG proteins are alterations to cellular mRNA transcription, splicing, stability, and translation. Such changes in cellular ribostasis could profoundly affect neural development and contribute to the devastating developmental and neurological anomalies observed following intrauterine ZIKV infection. Our study provides new insights into virus-host interactions and the identification of the SG proteins that may contribute to the unusual pathogenesis associated with this reemerging arbovirus.

## Abstract (precis)

Many viruses inhibit SGs. In this study, we observed that ZIKV restricts SG assembly, likely by relocalizing and subverting specific SG proteins to modulate ZIKV replication. This ZIKV-SG protein interaction is interesting, as many SG proteins are also known to function in neuronal granules, which are critical in neural development and function. Moreover, dysregulation of different SG proteins in neurons has been shown to play a role in the progression of neurodegenerative diseases. The likely consequences of ZIKV modulating SG assembly and subverting specific SG proteins are alterations to cellular mRNA transcription, splicing, stability, and translation. Such changes in cellular ribostasis could profoundly affect neural development and contribute to the devastating developmental and neurological anomalies observed following intrauterine ZIKV infection. Our study provides new insights into virus-host interactions and the identification of the SG proteins that may contribute to the unusual pathogenesis associated with this reemerging arbovirus.

## INTRODUCTION

Zika virus (ZIKV) is an enveloped, single-stranded positive-sense RNA virus belonging to the *Flaviviridae* family, which includes Dengue virus (DENV), yellow fever virus (YFV), and West Nile virus (WNV) (1). While ZIKV was discovered in Uganda in 1947 (2), the virus garnered renewed interest during the 2015 to 2016 outbreak in the Americas (3), in particular because of intrauterine infections and resulting developmental abnormalities, such as severe microcephaly, decreased brain tissue, macular scarring, congenital contractures, and hypertonia (4–9). Additionally, adults infected with ZIKV were reported to develop Guillain-Barré syndrome, a debilitating disorder affecting the peripheral nerves (10–13). Similar to other flaviviruses, ZIKV is transmitted by the Aedes aegypti and Aedes albopictus mosquitoes, although recent evidence has shown ZIKV is also capable of sexual and vertical transmission (14–17). While half a century has passed since the discovery of ZIKV, little to no research was published prior to the emergence of the current strain in the Americas associated with devastating developmental pathologies. Because there is no licensed vaccine and antiviral treatments are elusive, a fundamental understanding of the molecular biology of ZIKV and virus-host interactions is critical to developing therapeutic strategies.

The ZIKV single-stranded positive-sense RNA genome contains a 5ʹ-cap, lacks a poly(A) tail, and encodes one open reading frame (ORF) that is flanked by highly structured 5ʹ and 3ʹ untranslated regions (UTRs). Similar to other flaviviruses, translation of the ZIKV RNA results in one long polyprotein that is co- and posttranslationally proteolytically processed to produce at least three structural proteins (capsid [C], premembrane [prM], and envelope [E]) and seven nonstructural proteins (NS1, NS2a, NS2b, NS3, NS4a, NS4b, and NS5) (1). Although cap-dependent and cap-independent translation has been reported for DENV (18), it is presently unknown whether ZIKV employs similar translation strategies. Similarly, little is known regarding the strategies ZIKV employs to promote translation of the viral RNA.

To limit translation of viral RNAs or protect the cells from different environmental stresses, mammalian cells rapidly stall translation via the activation of one of the four eIF2α kinases. In particular, the presence of double-stranded RNA (dsRNA) during viral infection activates protein kinase R (PKR) (19) and the accumulation of unfolded proteins in the endoplasmic reticulum (ER), and resulting stress activates PKR-like endoplasmic reticulum kinase (PERK) (20), amino acid starvation activates general control nonrepressed 2 (GCN2) (21), and oxidative stress activates heme-regulated inhibitor kinase (HRI) (22). Phosphorylation of the α subunit of eIF2 by one of the four stress response kinases results in the stalling of translation initiation and disassembly of polysomes. Stalled translation initiation complexes bound to mRNA are recognized by several RNA binding proteins, which aggregate to form RNA-protein macromolecular complexes called stress granules (SGs) (23). Once the stressor is abated, eIF2α is dephosphorylated by protein phosphatase 1 (PPI) and the PPI cofactor growth arrest and DNA-damage-inducible 34 (GADD34), allowing for the return of sequestered mRNA transcripts to active translation (23).

SGs are dynamic non-membrane-bound cytoplasmic structures that can rapidly assemble in response to stress and disassemble once the stress has been alleviated (23). SGs typically contain mRNAs, stalled translation initiation complexes, and numerous RNA binding proteins. Indeed, SGs may contain upwards of 260 different proteins (24), and ∼50% of these are proposed to be RNA-binding proteins (25). Of these, Ras-GTPase activating binding protein 1 (or GAP SH3 domain binding protein 1 [G3BP1]), Caprin1, T-cell internal antigen 1 (TIA-1), and TIA-1-related protein (TIAR) are proposed to be key nucleators of SG assembly (26–29). In addition to translation repression and mRNA sorting, SGs also amplify the innate immune response by aggregating critical antiviral factors (23). Because translation is a critical first step in the flavivirus life cycle, the formation of SGs presents an immediate obstacle to infection. Notably, however, during infection with different flaviviruses, such as WNV, DENV, and Japanese encephalitis virus (JEV), SGs are absent, and treatment of virus-infected cells with arsenite fails to induce SGs (30–33). While WNV, DENV, and JEV all belong to the same flavivirus genus, each virus employs a unique mechanism to block SG assembly. For example, early during WNV infection PKR is activated by the appearance of exposed dsRNA replication intermediates, which results in phosphorylation of eIF2α, the stalling of translation initiation, and formation of stress granules. However, at later times during infection SG formation is limited as membranous vesicles mask dsRNA and viral replication complexes; thus, PKR remains inactive (30, 34, 35). More recently, WNV was shown to also limit SG assembly by upregulating and activating key transcription factors that modulate the antioxidant response (32). In contrast, p38-Mnk1 signaling and phosphorylation of the cap-binding protein eIF4E was affected during DENV infection, thereby inhibiting SG formation via an eIF2α phosphorylation-independent mechanism (33). Flaviviruses also subvert specific SG proteins to promote viral gene expression. For example, the SG-nucleating proteins TIA-1 and TIAR facilitate WNV replication (36); G3BP1, G3BP2, and Caprin1 promote translation of interferon-stimulated genes (ISGs) to limit DENV infection (37, 38); and JEV inhibits SG assembly by colocalizing Caprin1 with the viral capsid protein (31).

Similar to DENV and WNV, ZIKV was recently shown to limit the assembly of SGs (39, 40). In particular, Hou et al. reported that exogenously expressed Flag-tagged NS3 and NS4A repressed translation, and the formation of SGs was restricted when Flag-tagged capsid, NS3, NS2B-3, and NS4A proteins were individually expressed (39). Moreover, ZIKV RNA was bound by G3BP1, and G3BP1 and Caprin1 coimmunoprecipitated Flag-tagged ZIKV capsid protein (39). Depletion of TIAR, G3BP1, and Caprin1 decreased viral titers, likely as a result of disrupting the interactions with ZIKV capsid protein and RNA (39). Hou et al. also showed that SG assembly in ZIKV-infected cells was the consequence of eIF2α phosphorylation and translational repression by activating PKR and the unfolded protein response (39). Amorim et al. additionally reported that ZIKV limits the antiviral stress response by promoting an increase in the rate of eIF2α dephosphorylation during infection (40). While these studies provide insight into how SG formation is inhibited during ZIKV infection, the role of various SG proteins in ZIKV gene expression has yet to be elucidated.

A number of the RNA-binding proteins that localize in SGs, such as Ataxin-2, G3BP1, human antigen R (HuR), and TIA-1, are known to contribute to different neuropathologies (41, 42). Thus, our goal in this study was to investigate the biological impact of different SG proteins linked to neurodegeneration on ZIKV gene expression. Here, we show a systematic analysis of SGs during ZIKV infection and the effect of depleting six different SG proteins on ZIKV protein and RNA levels and viral titers, and we elucidate the biological function of two SG proteins on ZIKV gene expression. We determined that ZIKV disrupted sodium arsenite-induced SG assembly and that several SG markers colocalized with sites of ZIKV replication. Additionally, the SG protein G3BP1 is required for ZIKV gene expression, while HuR exhibited antiviral activity. Using an MR766 *Gaussia* Luciferase (GLuc) reporter genome, our studies revealed that G3BP1 and HuR specifically modulate ZIKV replication. This work advances our understanding of the interplay between ZIKV, the cellular stress response, and cellular RNA metabolism, and it demonstrates a role for specific RNA-binding proteins in ZIKV replication.

(This article was submitted to an online preprint archive [43].)

## RESULTS

### ZIKV inhibits arsenite-induced SG formation.

**FIG 1:**
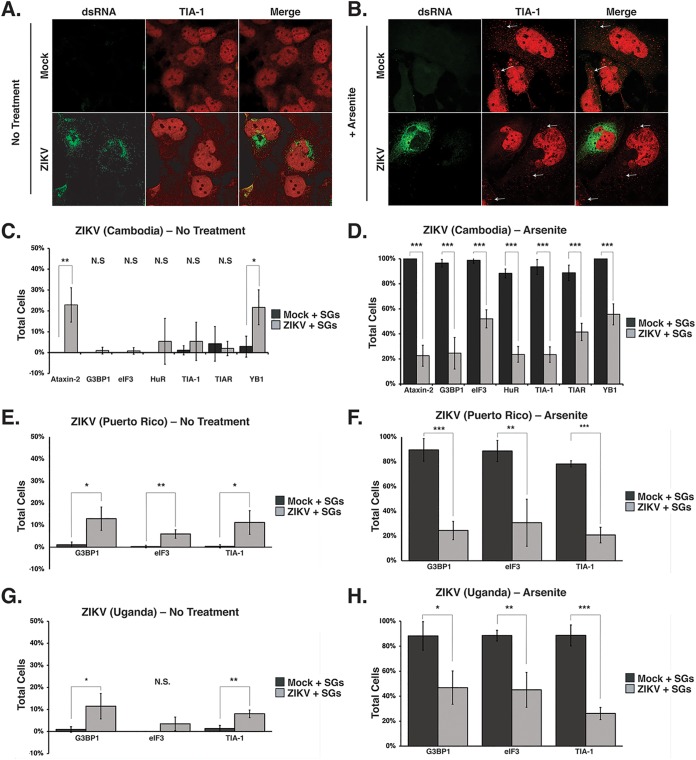
ZIKV restricts the assembly of cellular stress granules. Huh7 cells were either mock infected or infected with the Cambodia ZIKV strain (160310) at an MOI of 5. Twenty-four h postinfection, cells were mock treated or incubated with 1 mM sodium arsenite for 30 min, fixed, permeabilized, and analyzed using a 63× oil immersion objective on a Zeiss LSM710 laser scanning confocal microscope. ZIKV-infected cells were detected with mouse-anti-dsRNA antibody (green). Shown is the distribution of dsRNA (green) and the SG marker TIA-1 (red) in mock- and ZIKV-infected cells left untreated (A) or treated (B) with sodium arsenite. The white arrows highlight stress granules. (C and D) Quantification of SGs containing Ataxin-2, G3BP1, eIF3B, HuR, TIA-1, TIAR, and YB1 proteins in mock- and ZIKV (Cambodia 160310)-infected cells which were either not treated (C) or treated (D) with sodium arsenite. (E and F) The number of cells with G3BP1-, eIF3B-, and TIA-1-containing SGs in mock-infected cells or cells infected with a Puerto Rican isolate of ZIKV at an MOI of 5 and either not treated (E) or treated (F) with sodium arsenite. (G and H) SGs in mock-infected cells and cells infected with the original Ugandan isolate of ZIKV at an MOI of 5 were quantified. The number of cells containing G3BP1, eIF3B, and TIA-1-SG was quantified from cells that were untreated (G) or incubated with sodium arsenite (H). Note that in the original immunofluorescence images, TIA-1 was visualized using an anti-donkey Alexa Fluor 647 (magenta) anti-goat IgG secondary antibody. In panels A and B, TIA-1 has been pseudocolored red. The immunofluorescence images are representative of at least three independent experiments. The errors bars shown on the SG quantification data (C, D, E, F, G, and H) are means ± standard deviations (SD). Significance was determined by two-tailed Student *t* test (*, *P* < 0.05; **, *P* < 0.01; ***, *P* < 0.001; N.S., not significant).

To determine whether SGs form during ZIKV infection, Huh7 cells were infected with the Cambodian isolate (160310) at a multiplicity of infection (MOI) of 5, and the formation of SGs at 24 h postinfection was visualized by immunofluorescence and confocal microscopy using an antibody to detect TIA-1, a protein known to facilitate nucleation of SGs (27). ZIKV-infected cells were identified by staining for dsRNA, a marker of viral replication sites. In mock-infected cells, TIA-1 localized to the nucleus (Fig. 1A). Similar to other flavivirus-infected cells (30, 31), TIA-1 was mostly localized in the nucleus, and we observed ∼10% of ZIKV-infected cells containing SG foci in the cytoplasm (Fig. 1A and C). A lack of SGs during ZIKV infection suggested ZIKV either inhibited the formation of SGs or promoted the disassembly of SGs. To investigate whether ZIKV inhibited the formation of SGs, we treated cells with 1 mM sodium arsenite for 30 min. In mock-infected cells treated with sodium arsenite, we observed that 80% to 100% of the cells contained TIA-1 SGs in the cytoplasm (Fig. 1). In contrast, TIA-1-containing SGs were observed in ∼24% of ZIKV-infected cells treated with sodium arsenite (Fig. 1B and D).

We similarly investigated SG formation following infection with the 1947 Uganda isolate (MR766) and a recent ZIKV strain isolated in Puerto Rico in 2015 (PRVABC59) (44, 45). Similar to Huh7 cells infected with the Cambodia ZIKV strain (Fig. 1C), ∼10% of cells infected with MR766 or PRVABC59 had TIA-1-containing SGs (Fig. 1E and G). Consistent with the number of SGs in cells infected with the Cambodia ZIKV strain (Fig. 1D), approximately 20% of cells infected with MR766 or PRVABC formed TIA-1-SGs following treatment with sodium arsenite (Fig. 1F and H). These data indicate that the three ZIKV strains (Cambodia, Uganda, and Puerto Rico) examined restrict the formation of SGs when treated with sodium arsenite and are consistent with SG studies undertaken with WNV, DENV, and ZIKV (30, 32, 33, 39, 40).

To determine whether ZIKV inhibited a particular subset of SGs, we next investigated the formation of SGs containing Ataxin-2, G3BP1, eIF3B, HuR, TIAR, and YB1 in mock- and ZIKV (Cambodia 160310)-infected cells in the absence or presence of sodium arsenite (Fig. 1C and D). Quantification of the different SG proteins in mock-infected cells showed that less than 10% of untreated cells contained SGs, while SGs were visible in more than 90% of cells treated with sodium arsenite (Fig. 1C and D). In contrast, analysis of SGs as visualized using the different SG proteins in cells infected with the Cambodia ZIKV isolate (160310) showed that 1% to 25% of untreated cells and 18% to 56% of sodium arsenite-treated cells contained SGs (Fig. 1C and D). We noted that a significant number of ZIKV-infected cells formed Ataxin-2- and YB1-containing SGs in untreated cells, and that the number of cells with SGs containing YB1 increased following sodium arsenite treatment. Overall, ∼20% of ZIKV-infected cells showed sodium arsenite-induced SGs containing common SG components (Fig. 1D, F, and H). However, we also observed that 44% of infected cells showed SGs containing the translation initiation factor eIF3B (Fig. 1D, F, and H), indicating that the SGs induced in ZIKV-infected cells contain translationally repressed ribonucleoprotein complexes. Together these data show that all three ZIKV strains inhibit the formation of SGs, albeit to different extents.

### ZIKV does not dramatically change the abundance or integrity of SG proteins during infection.

**FIG 2:**
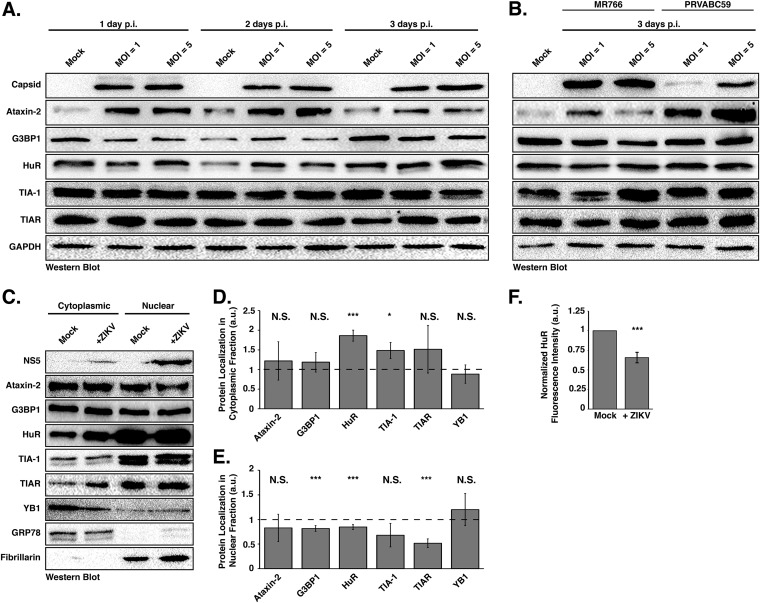
ZIKV infection changes the nuclear and cytoplasmic distribution of stress granule proteins. (A) Huh7 cells infected with the Cambodia strain of ZIKV at an MOI of 1 or 5 were harvested at 1, 2, and 3 days postinfection. Detection of ZIKV capsid protein by Western blotting confirmed viral infection. The abundance and integrity of Ataxin-2, G3BP1, HuR, TIA-1, and TIAR proteins were also examined at each time point. The Western blot shown is representative of at least three independent experiments. (B) Western blot analysis of SG proteins in MR766- and PRVABC59-infected Huh7 cells 3 days postinfection. (C) Cytoplasmic and nuclear distribution of Ataxin-2, G3BP1, HuR, TIA-1, TIAR, and YB1. Huh7 cells were mock infected or infected with the Cambodia strain of ZIKV at an MOI of 5, and 1 day postinfection the cytoplasmic and nuclear fractions were isolated. The distribution of ZIKV NS5, Ataxin-2, G3BP1, HuR, TIA-1, TIAR, YB1, GRP78, and Fibrillarin was analyzed by Western blotting. The Western blot shown is representative of the three independent experiments performed. (D and E) Quantification of the distribution of Ataxin-2, G3BP1, HuR, TIA-1, TIAR, and YB1 in the cytoplasmic (D) and nuclear (E) subcellular fractions. The abundance of each SG protein in the cytoplasmic or nuclear fraction was standardized against GRP78 or Fibrillarin, respectively. These values were then normalized against the abundance of the specific SG protein in mock-infected cells, which was assigned an arbitrary unit (a.u.) of 1 (denoted by dashed lines on each graph). The data presented in panels D and E were calculated from three independent infections, fractionations, and immunoblots. (F) Quantification of fluorescence intensity signal of the localization of HuR in the nucleus. Fluorescence intensity was derived in ImageJ using a freehand selection of the nucleus in mock- and ZIKV-infected cells. Values obtained for HuR were divided by the nucleus signal (Hoechst) within mock- and ZIKV-infected cells. Fluorescence intensity of the infected cells was standardized against that of mock-infected cells and arbitrarily assigned a value of 1. The relative fluorescence intensity signal was calculated from three independent experiments, and nine cells were counted per experiment. Two-tailed Student *t* tests were performed to calculate significance. *, *P* < 0.05; ***, *P* < 0.001; N.S., not significant.

To determine if the ability of ZIKV to inhibit sodium arsenite-induced SG assembly is a result of promoting proteolytic cleavage of and/or degrading SG-nucleating proteins, we infected Huh7 cells with ZIKV at MOIs of 1 and 5 and investigated the abundance and integrity of select SG proteins at 1, 2, and 3 days postinfection. By Western blot analysis we found that the abundance of most SG proteins remained the same between mock- and Cambodia ZIKV-infected samples (Fig. 2A), and no cleavage products were identified (Fig. 2A and B). Interestingly, independent of MOI, the levels of Ataxin-2 increased with ZIKV infection at 1 and 2 days postinfection (Fig. 2A). We similarly observed an increase in Ataxin-2 abundance and no change in HuR, TIA-1, and TIAR levels following infections with the Ugandan and Puerto Rican strains (Fig. 2B). We also observed a modest decrease in G3BP1 levels (Fig. 2A and B). While poliovirus 3C protease has been shown to cleave G3BP1 and block the formation of SGs, we did not observe such a cleavage product (46). It is possible that G3BP1 was cleaved during ZIKV infection; however, the modest decrease in G3BP1 levels likely limited the detection of such a fragment by Western blot analysis. Alternatively, G3BP1 might be degraded during ZIKV infection. Thus, the ability of ZIKV to block the assembly of stress granules was not the result of a change in the abundance or proteolytic cleavage of HuR, TIA-1, and TIAR. However, changes in Ataxin-2 and G3BP1 levels might in part restrict the formation of SGs during ZIKV infection.

### SG proteins are relocalized during ZIKV infection.

To investigate whether SG proteins are relocalized during ZIKV infection, we examined the relative distribution of different SG markers in the cytoplasm versus nucleus. In particular, Huh7 cells were either mock or ZIKV infected at an MOI of 5, and 1 day postinfection the cytoplasmic and nuclear fractions were isolated and the subcellular distribution of Ataxin-2, G3BP1, HuR, TIA-1, TIAR, and YB1 analyzed by Western blotting (Fig. 2C). To demonstrate effective isolation of the cytoplasmic and nuclear fractions and to show standardization of the amount of loaded proteins, we examined the localization and abundance of GRP78 (or BiP), an endoplasmic reticulum chaperone protein, and Fibrillarin, the methyltransferase protein that 2ʹO-methylates rRNA and is localized in the nucleoli. Western blot detection of ZIKV NS5 confirmed infection and, consistent with immunofluorescence studies (see Fig. 5A and B) (47), showed that ZIKV NS5 was predominantly localized in the nuclear fraction. We semiquantified the amount of the respective SG protein in each fraction by normalizing the band intensities in the cytoplasmic and nuclear fractions to GRP78 and Fibrillarin, respectively. Normalized values were then standardized to mock-infected samples to obtain a relative change in distribution of each protein. Thus, a ratio of band intensities of less than one indicates a decrease in the respective fraction, while values greater than one represent an enrichment of the SG protein in the subcellular fraction (Fig. 2D and E). Overall, we did not observe differences in the distribution of Ataxin-2, G3BP1, TIA-1, TIAR, and YB1 between the cytoplasmic and nuclear fractions (Fig. 2D and E). In contrast, however, the relative abundance of HuR in the nuclear fraction decreased, and the concomitant amount of HuR in the cytoplasm increased (Fig. 2D and E). Indeed, immunofluorescence analysis of HuR localization in mock- and ZIKV-infected cells showed strong nuclear staining and increased staining in the cytoplasm in ZIKV-infected cells (see Fig. 5B). We also quantified the fluorescence signal from confocal microscopy images by examining HuR localization in mock- and ZIKV-infected cells. In particular, the fluorescence signal in confocal microscopy images of HuR in the nucleus was quantified. The relative fluorescence intensity signal of HuR in mock-infected cells was arbitrarily set to 1 (Fig. 2F). Using this quantification analysis, a value greater than one indicates increased nuclear localization, and a value less than one indicates decreased localization in the nucleus. Similar to the subcellular fractionation and Western blot analysis, we observed a decrease in the amount of HuR protein in the nucleus in ZIKV-infected cells compared to that in mock-infected cells (Fig. 2E and F). These data together show that the subcellular localization of most SG proteins does not significantly change during ZIKV infection. In contrast, however, HuR is redistributed from the nucleus into the cytoplasm in ZIKV-infected cells. That SG proteins, particularly those that promote SG assembly, do not significantly accumulate in the cytoplasm may in part contribute to the reduction in the number of SGs during ZIKV infection.

### G3BP1 and HuR modulate ZIKV gene expression.

**FIG 3:**
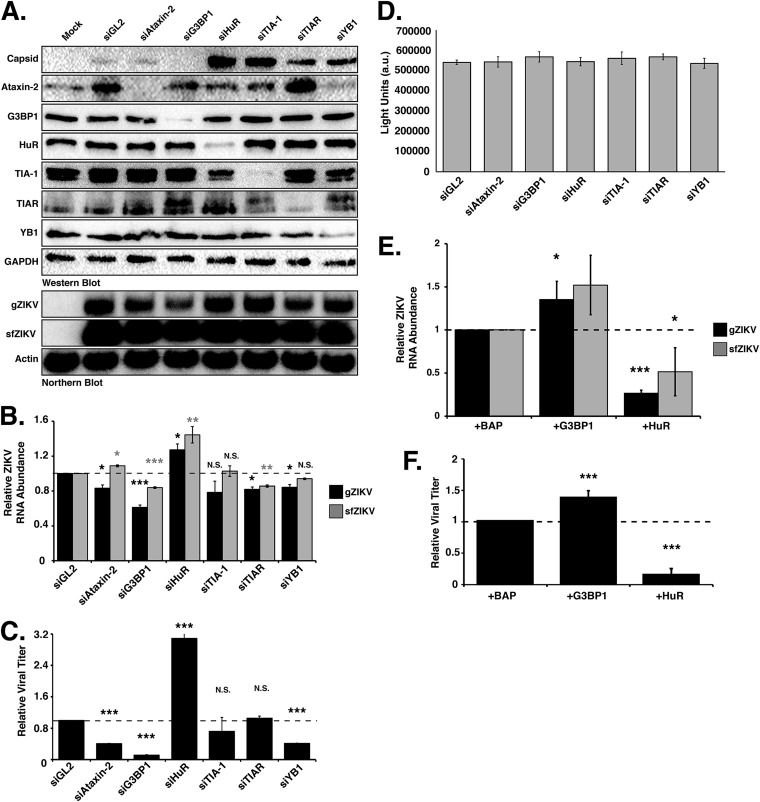
Stress granule proteins promote and limit ZIKV gene expression. Ataxin-2, G3BP1, HuR, TIA-1, TIAR, and YB1 proteins were depleted in Huh7 cells using target-specific siRNAs and then infected 24 h later at an MOI of 5 with the Cambodia strain of ZIKV (160310). An siRNA targeting *Gaussia* luciferase (siGL2) was used as a control, nontargeting siRNA. Protein, RNA, and media were harvested 48 h postinfection. (A) Western blot shows expression of ZIKV capsid and depletion of each stress granule protein. Expression of glyceraldehyde-3-phosphate dehydrogenase (GAPDH) was used as a loading control, and the Western blot shown is representative of at least three independent experiments. The Northern blot shows the effect of depleting each stress granule protein on the abundance of the ZIKV genomic (gZIKV) and subgenomic flaviviral (sfZIKV) RNA. Actin mRNA levels were evaluated as a loading control. The Northern blot shown is representative of at least three independent experiments. (B) Quantification of ZIKV gRNA and sfRNA. ZIKV RNA levels were normalized to actin mRNA and then represented relative to the siGL2 control. (C) Viral titers in the extracellular media were determined by plaque assay, and relative viral titers were calculated by normalizing titers relative to those of siGL2-transfected cells. (D) A representative CellTiter-Glo assay. Arbitrary light units (a.u.) quantify the effect of siRNA depletion of the six SG proteins on cell viability. (E) Quantification of ZIKV RNA levels following overexpression of G3BP1-Flag and HuR-Flag. Huh7 cells were transfected with plasmids expressing 3×Flag-bacterial alkaline phosphatase (BAP; control), G3BP1-Flag, or HuR-Flag and then infected with ZIKV (Cambodia 160310) 24 h posttransfection and then harvested 48 h postinfection. The effect of overexpressing G3BP1-Flag and HuR-Flag on gZIKV and sfZIKV RNA was evaluated by Northern blotting. (F) Effect of overexpression of G3BP1-Flag and HuR-Flag on viral titers. Data presented are from at least three independent experiments. Error bars show means ± SD, and statistical significance was determined using a two-tailed Student *t* test (*, *P* < 0.05; **, *P* < 0.01; ***, *P* < 0.001; N.S., not significant).

To elucidate the role of SG proteins during ZIKV infection, we transfected Huh7 cells with short interfering RNAs (siRNAs) targeting six different SG proteins and infected them with the Cambodian isolate (160310) at an MOI of 5. Two days postinfection, cells were harvested for protein and RNA analysis by Western and Northern blotting, and medium was collected for plaque assays. Western blot analysis from at least three independent experiments showed that the siRNAs effectively depleted the abundance of the targeted SG proteins (Fig. 3A). For the majority of the SG proteins examined, siRNA depletion of the individual SG proteins did not impact the levels of other SG markers. We did, however, observe that the levels of TIA-1 modestly decreased following knockdown of HuR (Fig. 3A). TIA-1 levels have similarly been shown to be modulated in cells deficient in another cellular RNA binding protein, namely, TIAR (36). In examining the effect of SG protein depletion on ZIKV, the abundance of the ZIKV capsid protein (Fig. 3A) did not change following depletion of Ataxin-2. We did, however, observe a dramatic decrease in viral protein when G3BP1 was depleted, while knockdown of HuR and TIA-1 substantially increased ZIKV capsid protein levels (Fig. 3A). Depletion of TIAR and YB1 similarly increased the amount of ZIKV capsid protein, albeit not to the same extent as depletion of HuR and TIA-1 (Fig. 3A).

We next examined the abundance of ZIKV genomic RNA (gRNA). By Northern blotting we observed significant reductions in ZIKV gRNA (gZIKV) levels following depletion of G3BP1 compared to those of cells transfected with the control siRNA, siGL2 (Fig. 3B). Depletion of Ataxin-2, TIAR, and YB1 also decreased gZIKV levels (Fig. 3B). Depletion of TIA-1 did not significantly affect the levels of viral RNA (Fig. 3B). Consistent with the increase in ZIKV capsid protein, depletion of HuR significantly increased gZIKV levels (Fig. 3B). During flaviviral infections, incomplete degradation of the viral gRNA by the cellular 5ʹ-to-3ʹ exonuclease Xrn1 produces a small noncoding viral RNA or subgenomic flavivirus RNA (sfRNA) that corresponds to the 3ʹ UTR (48). In our assays, we used a Northern blotting probe to the 3ʹ UTR and also detected the ZIKV sfRNA (Fig. 3A and B). Overall a decrease in ZIKV gRNA coincided with a decrease in sfRNA, although the abundance of sfRNA was greater than that of the gRNA, likely because both translating and newly replicated ZIKV RNA were degraded by Xrn1 (Fig. 3B).

We used plaque assays to elucidate the effect of depleting SG proteins on viral titers (Fig. 3C). While depletion of TIA-1 and TIAR had no effect on the production of infectious virus, depletion of Ataxin-2, G3BP1, and YB1 significantly reduced viral titers. Consistent with the increase in ZIKV protein and RNA following depletion of HuR, we also observed a significant increase in viral titers (Fig. 3C).

To ensure the effects on ZIKV following siRNA depletion of the SG proteins were not a result of a change in cell viability, we quantified ATP levels in metabolically active cells from Huh7 cells transfected with the respective siRNAs. The cell viability assay showed no significant differences in the metabolic activity in cells depleted of Ataxin-2, G3BP1, HuR, TIA-1, TIAR, and YB1, indicating that the changes in ZIKV protein, RNA, and viral titers were the consequence of knockdown of the specific SG protein (Fig. 3D).

Because siRNA depletion of G3BP1 and HuR showed the most dramatic effect on ZIKV gene expression, we chose to focus on these two SG proteins in subsequent experiments. To confirm the effect of G3BP1 and HuR on ZIKV gene expression, we overexpressed G3BP1-Flag and HuR-Flag in ZIKV-infected cells and examined viral RNA levels and viral titers (Fig. 3E and F). Analysis of viral RNA abundance showed that overexpression of G3BP1 and HuR significantly increased and decreased ZIKV RNA, respectively (Fig. 3E). Consistent with the effects on ZIKV RNA, we observed a corresponding increase and decrease in viral titers following overexpression of G3BP1 and HuR (Fig. 3F). Together these data indicate TIA-1, TIAR, and YB1 regulate translation of the ZIKV polyprotein and have a role in replication of the viral genome (Fig. 3A and B). The significant decrease in viral titer following depletion of YB1 further suggests a role for this cellular RNA-binding protein in virus assembly (Fig. 3C). Ataxin-2 and G3BP1 are proviral cellular factors, as depletion of Ataxin-2 and G3BP1 decreased ZIKV RNA levels (Fig. 3A and B) and overexpression of G3BP1 increased viral RNA (Fig. 3E), which in turn affected the production of infectious particles (Fig. 3F). Finally, HuR exhibits antiviral activity, as depletion of this RNA-binding protein significantly increased ZIKV protein and RNA levels and viral titers, and overexpression of HuR-Flag showed a reciprocal effect on ZIKV gene expression (Fig. 3).

### Changing the abundance of G3BP1 and HuR affects ZIKV replication.

**FIG 4:**
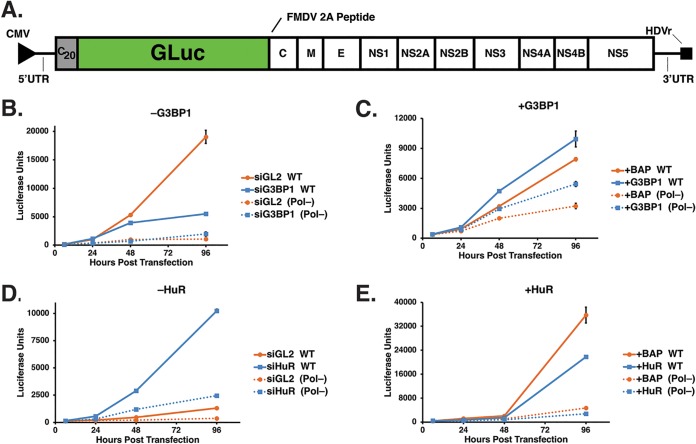
G3BP1 and HuR modulate ZIKV replication. (A) Schematic of the ZIKV MR766 *Gaussia* Luciferase reporter genome within the pCDNA6.2 MR766 clGLuc Intron3127 HDVr plasmid showing the 5ʹ and 3ʹ UTRs, the mature viral proteins within the single open reading frame, and the position of the *Gaussia* Luciferase gene within the MR766 genome. Two elements within the plasmid, namely, the CMV promoter and HDVr, which creates an authentic 3ʹ UTR in the genome, are also denoted. (B and D) Huh7 cells were first transfected with either the control or target-specific siRNAs and then transfected with the same siRNAs and the pCDNA6.2 MR766 clGLuc Intron3127 HDVr WT replication-competent or Pol(−) replication-deficient plasmid. GLuc activity in the medium of transfected cells was assayed at 6, 24, 48, and 96 h posttransfection. (C and E) Huh7 cells were transfected with p3×Flag-BAP, pG3BP1-Flag, pHuR-Flag, and WT and Pol(−) pCDNA6.2 MR766 clGLuc Intron3127 HDVr. At 6, 24, 48, and 96 h posttransfection, medium from the transfected cells was collected and GLuc activity measured. (B) Effect of G3BP1 knockdown on ZIKV-GLuc genome expression. (C) Effect on ZIKV-GLuc reporter genome expression following overexpression of 3×Flag-BAP (control) and G3BP1-Flag. (D) Effect of depleting HuR on ZIKV-GLuc genome expression. (E) Effect of overexpressing 3×Flag-BAP and HuR-Flag on ZIKV-GLuc genome expression. The data shown are from a single experiment and are representative of at least three independent experiments. Error bars indicate means ± SD.

Depletion of G3BP1 and HuR significantly decreased and increased ZIKV gene expression, respectively (Fig. 3), suggesting a role for these SG proteins in ZIKV translation and/or replication. To decipher the function of G3BP1 and HuR in ZIKV gene expression, we used the replication-competent (WT) and -deficient [Pol(−)] GLuc ZIKV reporter plasmid (pCDNA6.2 MR766 clGLuc Intron3127 HDVr) (Fig. 4A). These pCDNA6.2 MR766 clGLuc Intron3127 HDVr constructs were derived from the cDNA clone containing the full-length genome of the Uganda MR766 strain under the control of the cytomegalovirus (CMV) promoter (Fig. 4A) (49). Specifically, the full-length GLuc reporter gene, along with its signal sequence and the sequence for the foot-and-mouth disease virus (FMDV) 2A peptide, was cloned as a translational fusion following the first 20 amino acids of the capsid protein, and the full-length viral polyprotein was cloned downstream of GLuc. To avoid aberrant genome cyclization during replication due to duplicated 5ʹ cyclization elements, the flavivirus cyclization sequences in the full-length capsid coding region were disrupted by silent mutagenesis. Following transfection of this reporter construct, transcription and 5ʹ-end capping of ZIKV genomic RNA are directed by the host, and the hepatitis D virus ribozyme (HDVr) at the end of the 3ʹ UTR cleaves the ZIKV genomic RNA to create an authentic 3ʹ end (Fig. 4A) (49). Using GLuc expression as a proxy for viral RNA, translation of capped ZIKV RNA is detected 6 h posttransfection. Translation of the ZIKV polyprotein, including the synthesis of NS5, the RNA-dependent RNA polymerase (RdRp), directs replication of the reporter genome such that the increase in viral RNA as a result of replication is observed at 48 and 96 h posttransfection. We also expressed a ZIKV GLuc reporter genome that contained a mutation in the RdRp active site [GDD to GNN; pCDNA6.2 MR766 clGLuc Intron3127 Pol(−) HDVr], which rendered this mutant GLuc reporter genome replication deficient [Pol(−)]. Expression of the Pol(−) reporter genome showed a clear difference between replicating WT genomes and ZIKV RNA transcribed from the transfected plasmid at 48 and 96 h posttransfection (Fig. 4B to E).

To elucidate effects on translation and replication, we examined expression of the ZIKV GLuc reporter genome following siRNA depletion or overexpression of G3BP1 and HuR (Fig. 4B to E). RNA interference (RNAi) depletion or overexpression of G3BP1 and HuR did not significantly affect GLuc expression at 6 and 24 h posttransfection of both the ZIKV WT and Pol(−) reporter genomes (Fig. 4B to E), indicating that these two SG proteins do not affect translation of the ZIKV genome (Fig. 4B to E). At 96 h posttransfection of the WT reporter genome, however, siRNA depletion and overexpression of G3BP1 notably decreased and increased GLuc expression, respectively, showing that G3BP1 facilitates ZIKV replication (Fig. 4B and C). Curiously, overexpression of G3BP1 also increased GLuc expression from the ZIKV Pol(−) reporter genome (Fig. 4C). Since this ZIKV Pol(−) reporter genome is replication deficient, the increased levels of host-transcribed ZIKV mRNA might be the result of G3BP1 binding (39) and stabilizing the viral mRNA. In contrast to the effect of G3BP1, at 96 h posttransfection depletion of HuR dramatically increased GLuc expression (Fig. 4D). Reciprocally, overexpression of HuR decreased luciferase units more than expression of the 3×Flag-BAP control (Fig. 4E), indicating that HuR negatively impacts ZIKV replication. Taken together, these data support a role for G3BP1 and HuR in replication of the ZIKV genome.

### G3BP1 and HuR localize with ZIKV replication complexes.

**FIG 5:**
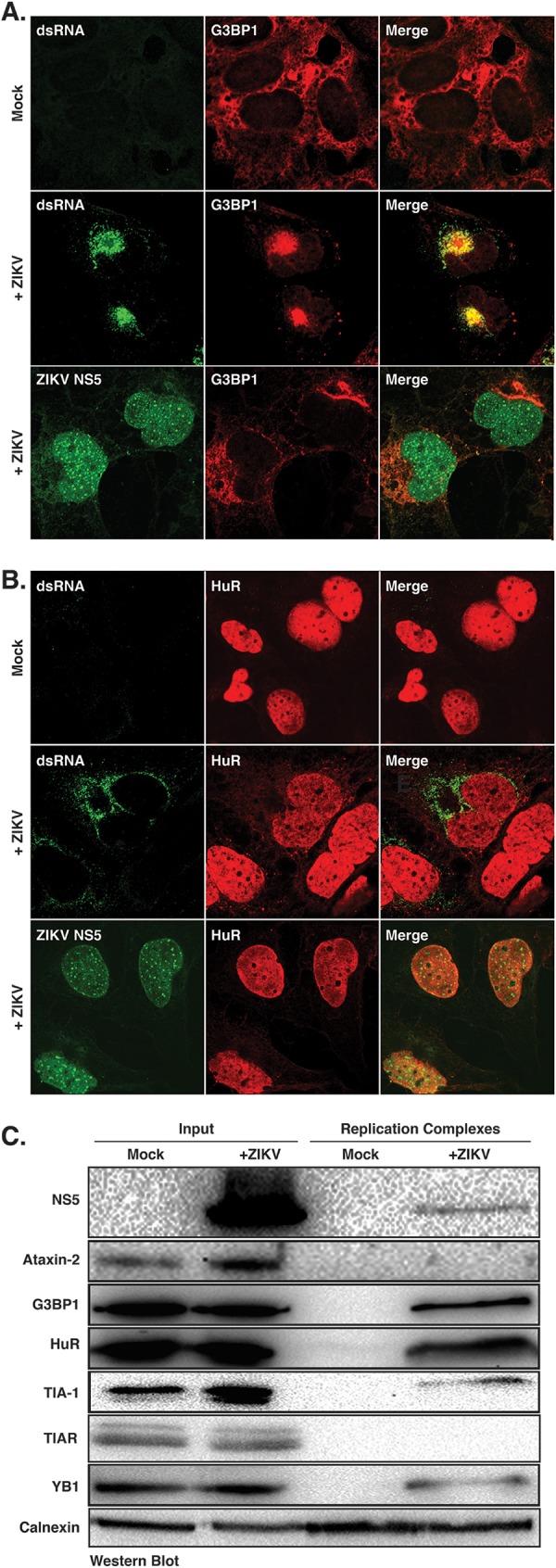
G3BP1 and HuR localize with ZIKV replication complexes. (A and B) Huh7 cells were either mock infected or infected with the Cambodia ZIKV strain at an MOI of 5. One day postinfection cells were fixed, permeabilized, and prepared for confocal imaging. Immunofluorescence images are representative of at least three independent experiments. (A) Immunofluorescence image showing G3BP1 (red) localization in mock-infected cells and with viral replication sites as visualized by staining with antibodies to dsRNA and NS5 (green) in ZIKV-infected cells. (B) Localization of HuR (red) in mock-infected cells and with dsRNA and NS5 (green) in ZIKV-infected cells. (C) Western blot analysis of ZIKV NS5, Ataxin-2, G3BP1, HuR, TIA-1, TIAR, YB1, and calnexin associated with replication complexes isolated from mock- and ZIKV-infected Huh7 cells. The Western blot is representative of three independent experiments.

To further demonstrate a role for G3BP1 and HuR in ZIKV replication, we next examined the localization of these two SG proteins at replication complexes (Fig. 5). We first examined the localization of G3BP1 and HuR at replication complexes by immunofluorescence analysis. In particular, we used antibodies to dsRNA and ZIKV NS5 protein as markers for replication sites (Fig. 5A and B). While dsRNA is a replication intermediate of negative- and positive-sense dsRNA, NS5 is critical for the replication of the ZIKV RNA, as it functions as the RdRp and the methyltransferase that caps the 5ʹ end of the ZIKV genome (1). G3BP1 shows diffuse cytoplasmic staining in mock-infected cells; however, in ZIKV-infected cells we observed strong colocalization of G3BP1 with dsRNA (Fig. 5A) and some colocalization with NS5 in the cytoplasm (Fig. 5A). In mock-infected cells, HuR was predominantly localized in the nucleus (Fig. 5B). Consistent with the subcellular fractionation data (Fig. 2B and C), we observed an increase in the localization of HuR in the cytoplasm of ZIKV-infected cells (Fig. 5B). Although HuR did not colocalize with dsRNA in ZIKV-infected cells, we did note HuR staining adjacent to ZIKV replication sites (Fig. 5B). HuR showed similar localization with NS5 in the cytoplasm (Fig. 5B). The localization of G3BP1 and HuR with respect to ZIKV replication sites supports a role in the synthesis of viral RNA.

Similar to other immunofluorescence studies (47), we observed strong nuclear and weak cytoplasmic localization for the ZIKV NS5 protein (Fig. 5A and B). Such localization was consistent with the cytoplasmic/nuclear fractionation data (Fig. 2B, C, and D). Because both NS5 and HuR are largely nuclear, we anticipated that these proteins might colocalize. Although immunofluorescence analysis showed diffuse nuclear staining for both NS5 and HuR, HuR did not colocalize with NS5 foci in the nucleus (Fig. 5B). Both HuR and flavivirus NS5 proteins have been shown to affect RNA splicing (50). While it is possible that HuR and NS5 together in the nucleus modulate processing of cellular RNAs, further interaction studies would need to be undertaken to verify such a role.

To corroborate the immunofluorescence analyses and role for G3BP1 and HuR in ZIKV replication, we also isolated replication complexes from mock- and ZIKV-infected Huh7 cells by sucrose gradient ultracentrifugation and then investigated the localization of select SG proteins with replication complexes. Western blot analysis showed the isolated fractions were derived from the endoplasmic reticulum, as shown by calnexin immunostaining in both mock- and ZIKV-infected fractions, and NS5 protein was only isolated in membrane fractions isolated from ZIKV-infected cells. G3BP1 and HuR were isolated with the replication complexes (Fig. 5C). While TIA-1, TIAR, and YB1 have previously been shown to affect WNV replication (30, 36), we only observed a small amount of TIA-1 and YB1 in the enriched membranes (Fig. 5C). While we noted an increase in the Ataxin-2 levels in ZIKV-infected cells (Fig. 2A), Ataxin-2 did not sediment with replication complexes (Fig. 5C). That G3BP1 and HuR were isolated with replication complexes (Fig. 5C) further indicates that G3BP1 and HuR function to modulate ZIKV replication (Fig. 4).

### G3BP1 colocalizes with ZIKV envelope protein.

**FIG 6:**
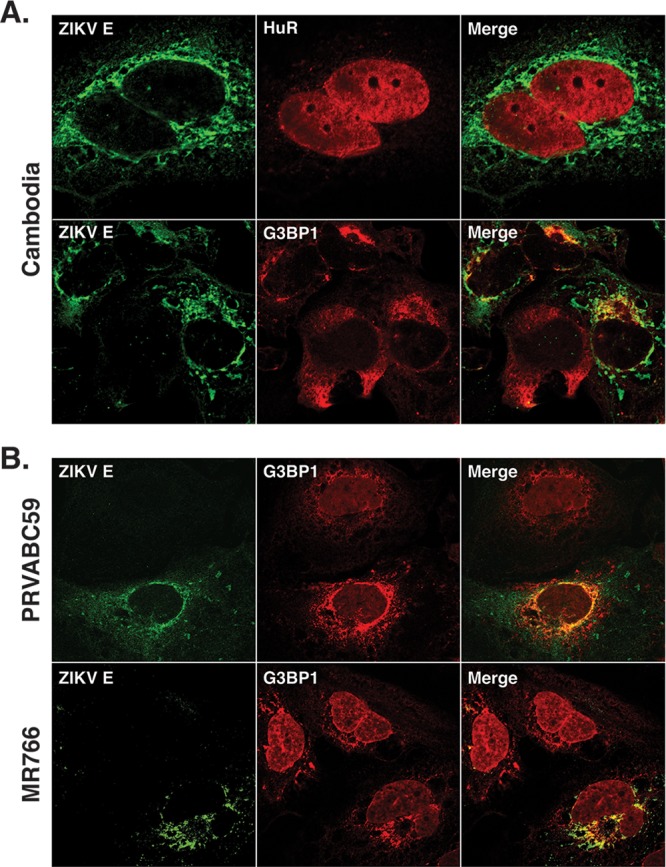
G3BP1 colocalizes with ZIKV E protein. One day postinfection, Huh7 cells infected with ZIKV at an MOI of 5 were fixed, permeabilized, and prepared for confocal imaging. (A) Localization of ZIKV E protein with HuR and G3BP1 following infection with the Cambodia strain of ZIKV. (B) Localization of ZIKV E protein with G3BP1 following infection with the Puerto Rican (PRVABC59) and Ugandan (MR766) strains of ZIKV. The immunofluorescence images shown are representative of at least three independent experiments.

Because ZIKV replication and assembly occur in close proximity on cellular membranes and G3BP1 was previously shown to interact with the capsid protein (39), we also investigated whether G3BP1 and HuR colocalized with ZIKV assembly sites. In particular, we examined the colocalization of both proteins with ZIKV envelope (E) protein by immunofluorescence analysis (Fig. 6). Similar to the localization of HuR with dsRNA and ZIKV NS5 protein (Fig. 5B), a small amount of HuR colocalized with ZIKV envelope protein (Fig. 6A). Interestingly, G3BP1 showed colocalization with the envelope protein in cells infected with the Cambodian ZIKV strain (Fig. 6A) and with the Puerto Rican (PRVABC59) and Ugandan (MR766) strains of ZIKV (Fig. 6B). The colocalization of G3BP1 with the ZIKV envelope protein together with the siRNA studies showing that depletion of G3BP1 modestly decreased ZIKV RNA by ∼40% (Fig. 3B), while viral titers were dramatically reduced (Fig. 3C), raises the possibility that G3BP1 functions more broadly than previously recognized. Specifically, G3BP1 promotes replication of the ZIKV genome and maybe also the assembly of new viral particles.

## DISCUSSION

The formation and sequestration of components of the translation machinery in SGs function as an intrinsic antiviral mechanism to restrict viral translation and/or replication. Flaviviruses have been shown to limit stress granule assembly by affecting cellular pathways. Specifically WNV was shown to modulate the cellular redox response by increasing the levels of the transcription factors that activate antioxidant genes and increase reduced glutathione levels (32). DENV modulates stress granule assembly by changing the phosphorylation of the cap-binding protein eIF4E by altering p38-Mnk1 signaling to affect cellular translation (33). More recently, ZIKV was shown to increase the rate of eIF2α dephosphorylation, which would promote translation and the rapid disassembly of stress granules (40). Interestingly, Hou et al. also showed that specific viral proteins impact cellular translation and the formation of stress granules (39). We similarly observed fewer SGs in ZIKV-infected cells and that the assembly of SGs following sodium arsenite treatment was inhibited during ZIKV infection (Fig. 1). Following depletion of TIAR, G3BP1, and Caprin-1, Hou et al. also found decreased ZIKV viral titers (39). In our study, we present a systematic investigation of the role of six different SG proteins on ZIKV protein and RNA abundance, production of infectious particles, colocalization with ZIKV, and effects on replication. Although G3BP1 from A549-infected cells coimmunoprecipitated ZIKV RNA (39), we specifically show that G3BP1 is in replication complexes (Fig. 5) and promotes synthesis of ZIKV RNA (Fig. 4B and C). Hou et al. also demonstrated that G3BP1 interacted with overexpressed Flag-tagged ZIKV capsid protein (39). We found that in ZIKV-infected cells, G3BP1 colocalized with ZIKV envelope protein, which raises the possibility that G3BP1 facilitates virion assembly in addition to its role in ZIKV replication (Fig. 6). Excitingly, we show that HuR functions to limit ZIKV replication, which to our knowledge has not previously been shown for this cellular RNA binding protein. Our systematic analysis of SG proteins during ZIKV infection is significant particularly as G3BP1, HuR, and other SG proteins have an intimate role in neurodevelopment and function. The modulation of SGs and SG proteins by ZIKV may have a more significant impact on normal gene regulation that contributes to ZIKV pathogenesis.

Ataxin-2 is a cytosolic SG protein that is associated with the neurodegenerative disorder spinocerebellar ataxia type-2 (SCA2) (51, 52). During ZIKV infection, we observed an increase in the abundance of Ataxin-2 (Fig. 2A). Furthermore, RNAi studies showed that depletion of Ataxin-2 did not affect ZIKV capsid levels (Fig. 3A) but did decrease the amount of viral RNA and viral titers (Fig. 3B and C). Ataxin-2 levels have been shown to affect the abundance of the poly(A) binding protein (PABP) (52), where elevated levels of Ataxin-2 decreased the amount of PABP (52). Such changes in PABP would be expected to negatively affect translation of cellular mRNAs and promote ZIKV gene expression. Moreover, Ataxin-2 interacts with the cellular RNA DEAD box helicase DDX6 (52), which we have previously shown to be required for ZIKV gene expression (53). Therefore, the impact on ZIKV RNA and production of infectious particles (Fig. 3B and C) following RNAi depletion of Ataxin-2 might also be the result of changes to DDX6. Analysis of SCA2 brains showed an increase in Ataxin-2 levels and that Ataxin-2 localized in intranuclear inclusions in 1% to 2% of neurons (54, 55). Our data showing that Ataxin-2 modulates ZIKV gene expression in the hepatocellular carcinoma cell line Huh7 is exciting, and additional studies in neuronal cells would illuminate the role of this protein in cellular dysfunction and neurodegeneration following intrauterine ZIKV infection.

The RNA-binding proteins TIA-1 and TIAR are broadly expressed in cells and tissues (56). These proteins are predominantly localized in the nucleus (Fig. 1 and data not shown), have been shown to modulate translation of specific cellular mRNAs (57), and are critical nucleators of stress granules in the cytoplasm (27). During WNV and DENV infection, TIA-1 and TIAR colocalize at replication sites and bind to the 3ʹ stem-loop region in the negative strand of WNV RNA to promote replication (30, 36, 58). We determined by Western blot analysis that TIA-1, but not TIAR, localized in isolated replication complexes (Fig. 5C). Because TIA-1 and TIAR were previously shown to promote WNV replication, we also investigated the role of TIA-1 and TIAR during ZIKV infection. Similar to Hou et al. (39), we found that decreasing the levels of TIA-1 had no effect on viral titers (Fig. 3C). In contrast to Hou et al. (39), we found that depletion of TIAR did not affect ZIKV titers (Fig. 3C). Our RNAi studies also demonstrated that following the reduction of TIA-1 and TIAR levels, the abundance of ZIKV capsid protein increased (Fig. 3A) and depletion of TIAR decreased ZIKV RNA levels (Fig. 3A and B), suggesting that, similar to tick-borne encephalitis virus, TIA-1 and TIAR are relocalized to perinuclear regions of replication to modulate translation of the ZIKV polyprotein (59). While the role of TIA-1 and TIAR in our studies in Huh7 differs from that in the study by Hou and colleagues, which were undertaken in A549 cells (39), the impact of TIA-1 and TIAR might be better elucidated in neuronal cells where a link between TIA-1, the microtubule protein Tau, and the neurodegenerative tauopathies has been described (60, 61).

Y box-binding protein-1 (YB1) modulates translation and stabilizes cellular mRNAs (57), and it also localizes in processing bodies and stress granules (62). YB1 has been shown to bind the DENV 3ʹ UTR to decrease DENV translation (63). In our study, siRNA depletion of YB1 increased the levels of ZIKV capsid protein (Fig. 3A), indicating that YB1, similar to its role in DENV infection, negatively regulates ZIKV translation. We also observed that YB1 is localized in replication complexes (Fig. 5C) and that depleting YB1 levels decreases ZIKV RNA levels (Fig. 3B). Notably, however, the modest effects of YB1 reduction on ZIKV protein and RNA levels differ from the significantly decreased viral titers (Fig. 3C). YB1 was previously shown to interact with the hepatitis C virus NS3/4A protein and other cellular factors to modulate the balance between replication and the assembly of HCV infectious particles (64, 65), and additional studies with YB1 may reveal a similar function during ZIKV infection.

G3BP1, similar to TIA-1, is an important nucleator of SGs (26, 28, 66). Interestingly, the role of G3BP1 varies in viral infections, from limiting alphavirus replication (67–69) to interacting with the HCV RdRp NS5B and enhancing the production of HCV infectious virus particles (70–72). In DENV-infected cells, G3BP1 interacts with the sfRNA (37, 73), and sequestration of G3BP1 by the sfRNA was shown to inhibit translation of interferon-stimulated genes (38). Hou et al. showed that siRNA depletion of G3BP1 decreased ZIKV titers and that G3BP1 also bound ZIKV RNA (39). In addition to the dramatic reduction in ZIKV titers following depletion of G3BP1 (Fig. 3C), we also observed decreased ZIKV protein and RNA levels (Fig. 3A and B). Together, these data suggested a role for G3BP1 in ZIKV translation, replication, and/or assembly. In our study we also used the ZIKV MR766 GLuc reporter genome (Fig. 4B and C) and showed a specific role in ZIKV replication, which was further supported by the localization of G3BP1 with ZIKV replication complexes (Fig. 5C) and colocalized with dsRNA (Fig. 5A). We also observed that ZIKV envelope protein (Fig. 6) colocalized with G3BP1, suggesting that in addition to facilitating replication of the ZIKV genome, G3BP1 has a role in assembly of new virus particles. Hou and colleagues report that G3BP1 binds ZIKV gRNA and that G3BP1 and Caprin-1 interact with the ZIKV capsid protein to increase ZIKV gene expression. While further mechanistic studies are required to demonstrate a role for G3BP1 in ZIKV assembly, it is possible that a multiprotein complex between ZIKV RNA, capsid protein, G3BP1, and Caprin-1 will shift the equilibrium from replication of the viral genome toward localization with the ZIKV envelope protein and the assembly of infectious ZIKV particles.

Human antigen R (HuR) is a member of the ELAVL RNA-binding protein family and is ubiquitously expressed (74). Notably, the three additional members of the ELAVL family (HuB/C/D) are highly expressed in the brain (74). HuR binding to poly(U)- and AU-rich elements within the 3ʹ UTR increases mRNA stability (75, 76). During infection with the single-stranded positive-sense alphaviruses Sindbis virus (SINV) and chikungunya virus (CHIKV), HuR was dramatically relocalized from the nucleus into the cytoplasm (77, 78). By subcellular fractionation and Western blot analysis, we similarly observed that HuR is redistributed into the cytoplasm (Fig. 2B), albeit not to the same extent as that during alphavirus infection (77, 78). HuR is recruited to alphavirus RNA via the U-rich elements within the 3ʹ UTR, and this interaction stabilizes and protects the viral RNA from the cellular mRNA decay machinery (78). Although HuR has been shown to promote alphavirus mRNA stability (77, 78) and HCV internal ribosome entry site-mediated translation (70, 79), our study indicates an antiviral role for HuR during ZIKV infection (Fig. 3), which to our knowledge has not previously been shown. In particular, we found that HuR modulates ZIKV replication (Fig. 4D and E), which resulted in increased ZIKV protein and RNA levels and viral titers (Fig. 3). The role of HuR in ZIKV replication was further supported by the detection of HuR in isolated replication complexes (Fig. 5C). Curiously, immunofluorescence studies showed that HuR in the cytoplasm was localized adjacent to dsRNA and ZIKV NS5 protein staining (Fig. 5B). We note that the localization of HuR in replication complexes, when analyzed in isolated replication complexes versus immunofluorescence analysis, does not strongly correlate. However, it is important to highlight that HuR is strongly localized in the nucleus (Fig. 5B and 6A), and this nuclear staining likely overshadows the relocalization of HuR into the cytoplasm and replication complexes. Indeed, immunofluorescence analysis of ZIKV NS5 protein similarly showed strong nuclear and weak cytoplasmic localization (Fig. 5B), despite being the RdRp responsible for replicating the ZIKV RNA genome. A role for HuR in ZIKV replication was further supported by siRNA depletion of HuR and overexpression of HuR-Flag that increased and decreased, respectively, replication of MR766 GLuc reporter expression (Fig. 4D and E). The mode by which HuR limits ZIKV replication remains to be elucidated. A bioinformatic analysis of the ZIKV 3ʹ UTRs identified putative HuR binding sites (data not shown), raising the possibility that, similar to the case for SINV (78), HuR interacts with ZIKV RNA to alter the subcellular localization of HuR and directly affects ZIKV replication, possibly by destabilizing, rather than stabilizing, the viral RNA. Alternatively, the consequence on ZIKV replication might be indirect, where HuR relocalization affects cellular RNA homeostasis or ribostasis. Indeed, relocalization of HuR by SINV into the cytoplasm was shown to decrease cellular mRNA stability and alter mRNA splicing and nuclear polyadenylation (80). Interestingly, another cellular RNA-binding protein, Fragile X mental retardation factor (FMRP), was shown to bind ZIKV sfRNA and limit viral infection (81). It will be interesting to determine the mechanism by which HuR functions during ZIKV and whether this regulatory mode is similar to that of FMRP. Such studies will provide new insights into virus-host interactions, particularly for RNA-binding proteins that function as restriction factors.

Beyond aggregating stalled translation complexes, RNA binding proteins in stress granules also localize in neuronal granules. These granules regulate neuronal growth and synaptic plasticity (82, 83). Interestingly, a number of stress granule proteins, such as Ataxin-2, G3BP1, TIA-1, and HuR, are known to contribute to different neuropathologies (41, 83). While transcriptional changes have been reported to contribute to the neurological and developmental defects observed following intrauterine ZIKV infection (84–87), the role of stress granules and granule proteins should not be overlooked. By limiting the formation of stress granules and relocalizing and subverting specific stress granule proteins, alterations in RNA homeostasis, such as changes in RNA splicing, RNA stability, and translation, could also contribute to ZIKV neuropathologies. To this end, elucidating the interactions of stress granule proteins and the cellular consequences, particularly in neuronal cells, could provide new insights into the pathomechanisms underlying ZIKV congenital disease.

## MATERIALS AND METHODS

### Cell maintenance and ZIKV stocks.

The hepatocellular carcinoma cell line Huh7 was maintained in Dulbecco’s minimal essential medium (DMEM; Life Technologies) supplemented with 10% fetal bovine serum (FBS; Seradigm), 10 mM nonessential amino acids (NEAA; Life Technologies), and 5 mM l-glutamine (Life Technologies). Vero cells (ATCC CRL-81) were maintained in DMEM supplemented with 10% FBS and 10 mM HEPES (Life Technologies). Mammalian cell lines were grown at 37°C with 5% CO_2_. C6/36 cells (ATCC CRL-1660) grown at 27°C with 5% CO_2_ were maintained in Eagle’s minimal essential medium (EMEM; Sigma) supplemented with 10% FBS, sodium pyruvate (0.055 g/liter; Life Technologies), amphotericin B (Fungizone) (125 μg/liter; Life Technologies), and penicillin and streptomycin (50,000 U/liter penicillin, 0.05 g/liter streptomycin; Life Technologies). ZIKV (Cambodia 160310, Uganda MR766, and Puerto Rico PRVABC59; generous gifts from Brett Lindenbach, Yale School of Medicine; Laura Kramer, Wadsworth Center NYDOH; and the CDC) stocks were generated in C6/36 cells. Briefly, C6/36 cells nearing confluence were infected at an MOI of 0.1. Seven days postinfection, supernatants from the infected cells were collected, aliquoted, and stored at −80°C. RNA was extracted from cells to confirm infection via Northern blotting, and viral titers were determined by plaque assay.

### siRNA and DNA plasmid transfections.

Sense and antisense siRNA oligonucleotides were synthesized by Integrated DNA Technologies (IDT). The siRNA sequences are provided in Table 1. Oligonucleotides were resuspended in RNase-free water to a 50 μM final concentration. Sense and antisense strands were combined in annealing buffer (150 mM HEPES [pH 7.4], 500 mM potassium acetate, and 10 mM magnesium acetate) to a final concentration of 20 μM, denatured for 1 min at 95°C, and annealed for 1 h at 37°C (70).

**TABLE 1:**
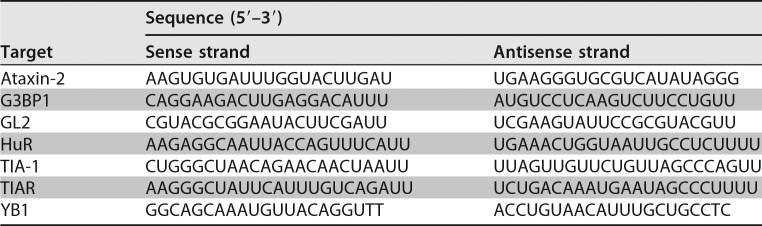
Sequences of siRNA oligonucleotides

For siRNA and plasmid transfections, Huh7 cells seeded at 7.5 × 10^6^ in a 10-cm cell culture dish were transfected 24 h later with 100 nM the indicated siRNA duplex or 2 μg of 3×FLAG-BAP (bacterial alkaline phosphatase; C7472; Sigma), HuR-Flag (OHu23723D; GenScript), or G3BP1-Flag (OHu02150D; GenScript) plasmids using Lipofectamine 3000 (Invitrogen) per the manufacturer’s protocol. At 24 h posttransfection, cells were infected with ZIKV.

### ZIKV infections.

On the day of infection, one mock culture plate was trypsinized and counted to calculate MOI. Cells were infected at an MOI of either 1 or 5, as noted in the text and figure legends. Appropriate amount of viral stocks thawed at room temperature were diluted in PBS to a final volume of 3.5 ml. Culture dishes were aspirated, the viral solution was added, and cells were returned to the incubator for 1 h with rocking every 15 min. At the end of the hour, 4 ml complete medium was added to each plate.

### Immunofluorescence analysis and confocal microscopy.

Cells seeded in 8-chambered slides (Nunc Lab-Tek chamber slide system; C7182; Sigma) were infected with ZIKV at an MOI of 5. Two days postinfection, cells were either mock treated or treated with sodium arsenite (1 mM final concentration; Sigma) for 30 min. Hereafter, the cells were washed three times with phosphate-buffered saline (PBS) and fixed with 4% paraformaldehyde–PBS for 10 min at room temperature. Cells were washed once with PBS and permeabilized with 100% ice-cold methanol for 15 min at room temperature. Cells were washed in blocking buffer (PBS–1% fish gelatin; Sigma) three times for 15 min. Primary antibody was diluted in blocking buffer, added to the appropriate wells, and incubated overnight at 4°C. Secondary antibodies diluted in blocking buffer were added for 1 h at room temperature in the dark. Hoechst-33342 (Life Technologies) was applied for 15 min. Between and after application of antibodies, cells were washed with blocking buffer three times for 15 min. Finally, the cells were washed twice with PBS for 5 min, the 8-chamber upper structure was removed, Fluoromount (Southern Biotech) was added, and a cover-slip was applied. Slides were stored at 4°C if imaging was undertaken within 1 week or at −20°C for long-term storage. Antibodies and concentrations used are listed in Table 2. Slides were imaged on a Zeiss LM710 confocal microscope with a 63× oil objective.

**TABLE 2:**
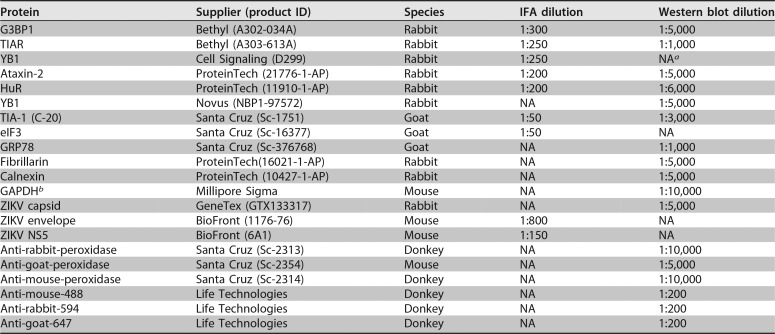
Antibodies used for immunofluorescence and Western blot analyses

*^a^*NA, not applicable.

*^b^*GAPDH, glyceraldehyde-3-phosphate dehydrogenase.

### Quantification of SG-positive cells.

A 40× oil objective was used to acquire images of ZIKV-infected cells stained with mouse-anti-dsRNA antibody to detect ZIKV-infected cells and one of the following anti-SG protein antibodies: rabbit anti-Ataxin-2, rabbit anti-G3BP1, rabbit anti-HuR, goat anti-TIA-1, rabbit anti-TIAR, and rabbit anti-YB1. To quantify the effect of ZIKV infection on SG formation, 200 to 300 infected cells between three or more biological replicates were counted. Quantification of SGs between different infections was completed as follows. For each treatment, the percentage of uninfected cells with/without SGs and ZIKV-infected cells with/without SGs was calculated. Using the percentages from cells with/without SGs from three or more biological replicates, a one-tailed Student *t* test was carried out.

### Subcellular fractionation.

Huh7 cells seeded in 10-cm cell culture dishes at 7.5 × 10^6^ were infected at an MOI of 5. Two days postinfection, the cells were trypsinized, centrifuged at 500 × *g* for 5 min, and resuspended in PBS. Cytoplasmic and nuclear proteins were separated using the NE-PER extraction reagents per the manufacturer’s protocol (ThermoScientific). After clarification of proteins, 30 μg of protein lysate from the cytoplasmic and nuclear fractions was analyzed by Western blot analysis. To demonstrate successful separation of cytoplasmic and nuclear fractions, the Western blots were also probed for the ER chaperone protein GRP78 and Fibrillarin, a methyltransferase enzyme that 2ʹO-methylates rRNA. Quantification of band intensities was performed using Image Lab software (Bio-Rad). The subcellular distribution of SG proteins in ZIKV-infected cells was normalized to the corresponding mock-infected lane, and an average of three independent experiments was used to generate the quantification graph.

### Harvest of ZIKV-infected cells for protein and RNA analysis.

At the indicated time point, media from culture dishes were aspirated and the cells were washed once with PBS. Cells were manually scraped from the plate in 1 ml PBS, divided between two microcentrifuge tubes, and pelleted at 14,000 rpm for 15 s. The supernatant was removed, and cells were resuspended in 50 μl radioimmunoprecipitation assay (RIPA) buffer (100 mM Tris-HCl [pH 7.4], 150 mM NaCl, 1% sodium deoxycholic acid, 1% Triton X-100, 0.1% SDS) containing protease inhibitors (mini tablets, EDTA-free; ThermoScientific Pierce) or 1 ml TRIzol (Invitrogen) for protein and RNA analysis, respectively. For protein analysis, cells resuspended in RIPA buffer with protease inhibitors were incubated on ice for 20 min. The lysate was clarified by centrifugation at 14,000 rpm at 4°C for 20 min. The clarified protein lysate was collected and processed for Western blot analysis. RNA from cell lysates was extracted using TRIzol per the manufacturer’s instructions.

### Western blot analysis.

Protein lysates were quantified using the DC protein assay kit (Bio-Rad), and 20 μg lysate was electrophoresed for 2 h at 100 V through SDS–10% PAGE. Proteins were transferred to a polyvinylidene difluoride membrane at 100 V for 60 min at 4°C. Transfer efficiency was determined by Ponceau S (Sigma) staining, after which the blots were washed in buffer (0.1% Tween in PBS [PBS-T]) and blocked for 30 min in blocking buffer (5% [wt/vol] powdered milk in PBS-T). Primary antibodies diluted in blocking buffer were added to the blots and incubated at 4°C overnight. Secondary antibodies diluted in blocking buffer were incubated at room temperature for 1 h. The assay was developed using Clarity Western ECL blotting substrate (Bio-Rad) and imaged on a chemiluminescent imager (Bio-Rad). Prior to application of additional antibody, blots were stripped using ReBlot Plus mild (Millipore Sigma) according to the manufacturer’s suggestions. Before and after application of primary/secondary antibodies and stripping, blots were washed for 15 min three times in wash buffer. Antibodies and concentrations are listed in Table 2.

### Northern blot analysis.

TRIzol-extracted RNA (10 μg) was resuspended in loading buffer (1× morpholinepropanesulfonic acid-EDTA-sodium acetate [MESA; Sigma], 4.5% formaldehyde, and 32% formamide), denatured for 15 min at 65°C, separated in a 1.2% agarose gel containing 5.92% (vol/vol) formaldehyde and 1× (vol/vol) MESA, and transferred via capillary action to a Zeta-probe membrane (Bio-Rad) overnight at room temperature. RNA was cross-linked to the membrane using a TL-2000 (UVP) and then stained with methylene blue to visualize transfer of RNA. Methylene blue was removed by washing the membranes in 1× SSC (1× SSC is 0.15 M NaCl plus 0.015 M sodium citrate)–1% SDS for 15 min three times and then prehybridized in 5 ml ExpressHyb (ClonTech) for 1 h at 65°C. The hybridization buffer was changed, and a ^32^P-labeled dsDNA probe (Invitrogen) was added for 1 h at 65°C. Blots were then washed with 0.1× SSC–0.1% SDS three times for 15 min at 55°C, exposed overnight to a phosphorimager screen, and subsequently visualized on a Typhoon 9400 (GE). The ZIKV 3ʹ UTR probe (targeting nucleotides 10324 to 10808 of the viral genome) was generated by reverse transcription-PCR amplification of the 3ʹ UTR from ZIKV-infected cells, and the resulting PCR product was cloned into pCR-TOPO2.1 (Invitrogen). The actin and ZIKV probes were randomly labeled with [α-^32^P]dATP (Perkin Elmer) using a RadPrime labeling kit (Invitrogen).

### Isolation of ZIKV replication complexes.

A protocol adapted from Schlegel et al. and Chen et al. was used to isolate ZIKV replication complexes (44, 45). Huh7 cells seeded in 15-cm cell culture dishes were either mock infected or infected with ZIKV (Cambodia 160310) at an MOI of 1 and then harvested 1 day postinfection. Specifically, cells were washed twice with cold PBS, gently dislodged using a cell lifter, collected, and pelleted at 1,000 rpm for 5 min at 4°C. The cell pellets were resuspended in 1 ml hypotonic buffer (10 mM Tris-HCl [pH 8.0], 10 mM NaCl, 1 mM MgCl_2_) containing protease and phosphatase inhibitors (mini tablets, EDTA-free; ThermoScientific Pierce). A one-tenth volume of cells (100 μl) was collected for the protein input sample, which was lysed in RIPA buffer as described above. The cells in hypotonic buffer were incubated on ice for 10 min. After incubation, cells were homogenized using the tight pestle of a Dounce homogenizer approximately 50 times on ice. Samples were then centrifuged at 1,000 × *g* for 10 min at 4°C, the supernatant was collected, and NaCl was added to the supernatants to a final concentration of 300 mM. The replication complexes in the collected supernatant were separated in a 10%/60% gradient. The 10%/60% sucrose gradient was prepared in Ultraclear tubes (Beckman Coulter) by first adding 5.5 ml of 10% sucrose solution (300 mM NaCl, 15 mM Tris-HCl [pH 7.5], 15 mM MgCl_2_, and 10% sucrose), followed by a slow deposition of 5.5 ml 60% sucrose (300 mM NaCl, 15 mM Tris-HCl [pH 7.5], 15 mM MgCl_2_, and 60% sucrose) below the 10% sucrose layer. Samples were layered onto the sucrose gradients and ultracentrifuged using an SW41 rotor at 26,000 rpm for 16 h at 4°C. The opaque layer sedimenting between the 10% and 60% sucrose layers was collected for analysis. For Western blot analysis, the proteins were precipitated with methanol and resuspended in a buffer containing 8 M urea and 100 mM Tris-HCl (pH 8.0).

### Plaque assays.

Six-well plates were seeded with 6 × 10^5^ Vero cells per well. The following day, serial dilutions of virus were prepared in PBS, medium was aspirated, and 300 μl of appropriate dilution was added. Cultures were returned to the incubator for 1 h with rocking every 15 min. After incubation, 2.5 ml of a 1:1 overlay (1.2% Oxoid agar and modified medium [2× DMEM, 4% FBS, 10 mM HEPES]) was added to each well. Agar was left to solidify at room temperature for 10 min before returning the plates to the incubator. At 4 days postinfection, plaques were developed using 1% crystal violet in 20% methanol.

### Cell viability assays.

Huh7 cells seeded at 5 × 10^5^ in a 6-cm cell culture dish were transfected 24 h later with the indicated siRNA duplex using Lipofectamine 3000 (Invitrogen) per the manufacturer’s protocol. After 24 h, cells were trypsinized and seeded in triplicate into a white 96-well plate. Forty-eight h after seeding the multiwell plate, the original 6-cm culture dish was harvested for protein to confirm knockdown, and a cell viability assay was performed. An equal volume of CellTiter-Glo 2.0 (100 μl; Promega) in cell culture medium was added to each well, which was rocked for 2 min following a 10-min room temperature incubation. Luminescence was recorded with an integration time of 1 s using a BioTEK Synergy luminometer.

### Luciferase reporter virus and assays.

To construct a ZIKV reporter virus, the *Gaussia* Luciferase gene was cloned as a fusion with the amino terminus of the polyprotein, as previously described for another ZIKV strain (88), into the previously described plasmid pCDNA6.2 MR766 Intron3127 HDVr, encoding a CMV promoter-driven MR766 ZIKV (49). Specifically, a translational fusion was generated comprised of the first 20 amino acids of the capsid protein, the full-length *Gaussia* Luciferase reporter gene along with its signal sequence, the FMDV 2A peptide, and finally the full-length viral polyprotein. The flavivirus cyclization sequence determinants in the full-length capsid coding region was disrupted by silent mutagenesis (indicated here by lowercase nucleotides: 5ʹ ATT GTa AAc ATG tTA AAA). To create a replication-incompetent version of this plasmid, this reporter cassette was also cloned into the previously described pCDNA6.2 MR766 Intron3127 Pol(−) HDVr (49) to create pCDNA6.2 MR766 clGLuc Intron3127 Pol(−) HDVr.

Huh7 cells were seeded into 6-cm cell culture dishes at a density of 5 × 10^5^ cells/dish. For siRNA experiments, the cells were transfected with siRNAs 24 h after seeding of the cells. Twenty-four h posttransfection, cells were subsequently seeded into 24-well plates at a density of 5 × 10^4^ cells/well. The following day, 200 ng of the replication-competent and replication-incompetent pCDNA6.2 MR766 clGLuc Intron3127 HDVr, along with appropriate siRNAs (40 nM) or plasmid DNA (500 ng), were transfected using Lipofectamine 3000. At 6, 24, 48, and 96 h posttransfection, media from wells were collected and stored at −20°C. Assays were performed using the NEB BioLux GLuc kit (E3300L) by following the stabilized protocol. Briefly, samples and GLuc assay solution were prepared and equilibrated to room temperature. The sample (35 μl) was added to three individual wells in a white 96-well plate. The GLuc assay solution (50 μl) was dispensed into each sample well, shaken for 5 s, and incubated at room temperature for 30 s, and luminescence was recorded with an integration time of 10 s using a BioTEK Synergy luminometer.

### Statistical analysis.

For RNA quantification of Northern blots, ImageQuantTL was used to obtain the mean density of gZIKV, sfRNA, and actin RNA. RNA levels of ZIKV were initially divided by the mean density of actin and subsequently standardized to the control siGL2 (or 3×Flag-BAP). A two-tailed Student *t* test comparing control RNA levels to those of each knockdown (or overexpression) sample was performed using data from three or more biological replicates. Relative viral titers were determined by standardizing the PFU/ml for each treatment to the control siRNA (siGL2) or plasmid (p3×FLAG-BAP). A two-tailed Student *t* test was performed using relative viral titers from three biological replicates. All graphs were generated using Microsoft Excel for Mac 2011. Statistical analysis was performed using StatPlus:mac LE.
